# DNA-protein quasi-mapping for rapid differential gene expression analysis in non-model organisms

**DOI:** 10.1186/s12859-024-05924-1

**Published:** 2024-10-24

**Authors:** Kyle Christian L. Santiago, Anish M. S. Shrestha

**Affiliations:** 1 Bioinformatics Lab, Advanced Research Institute for Informatics, Computing, and Networking, De La Salle University Manila, 2401 Taft Avenue, Manila, Philippines; 2 Department of Software Technology, College of Computer Studies, De La Salle University Manila, 2401 Taft Avenue, Manila, Philippines

**Keywords:** Quasi-mapping, DNA-protein alignment, RNA-seq, Non-model organism, Differential gene expression analysis

## Abstract

**Background:**

Conventional differential gene expression analysis pipelines for non-model organisms require computationally expensive transcriptome assembly. We recently proposed an alternative strategy of directly aligning RNA-seq reads to a protein database, and demonstrated drastic improvements in speed, memory usage, and accuracy in identifying differentially expressed genes.

**Result:**

Here we report a further speed-up by replacing DNA-protein alignment by quasi-mapping, making our pipeline > 1000× faster than assembly-based approach, and still more accurate. We also compare quasi-mapping to other mapping techniques, and show that it is faster but at the cost of sensitivity.

**Conclusion:**

We provide a quick-and-dirty differential gene expression analysis pipeline for non-model organisms without a reference transcriptome, which directly quasi-maps RNA-seq reads to a reference protein database, avoiding computationally expensive transcriptome assembly.

## Background

Due to decreasing cost of sequencing, RNA-seq has become a mainstay of gene expression studies on a wide variety of organisms spanning the tree of life. A vast majority of these organisms do not have well-annotated reference genomes or transcriptomes, which are required by standard RNA-seq data analysis pipelines. A conventional work-around for such “non-model” organisms has been to assemble a transcriptome sequence from the RNA-seq reads, which is then used as reference. Functional inference of differentially expressed genes are done by aligning the assembled contigs to reference protein databases to find orthologs.

In some applications of RNA-seq in non-model organisms, transcriptome assembly, which requires massive computational resources, tends to be an overkill. This is especially the case when the main aim is to identify over or under-expressed genes, and not, say, to discover novel transcripts. In such cases there is no direct interest nor enough sequencing depth to reconstruct good quality transcript sequences. Apart from being resource hungry, transcriptome assembly is also known to introduce errors such as over-extension, mis-assembly, etc [1, 2].

In our prior work, we proposed the novel pipeline SAMAR [3], which directly aligns RNA-seq reads to a protein database—the one that would have been after-all used by the assembly-based approach for functional analysis—to measure expression and for subsequent differential expression analysis. Another recently proposed software tool Seq2Fun [4] also uses nucleotide-to-protein alignment for gene abundance quantification and functional profiling of RNA-seq data. An obvious outcome of this direct alignment approach is the drastic reduction of computational costs compared to the assembly-based approach. More interestingly, we showed that this approach has significantly higher precision and recall than assembly-based approach in identifying differentially expressed genes.

Here we sought to further speed up SAMAR, by exploiting the fact that gene expression levels can be estimated based on just the knowledge of which reference sequence(s) each read maps to, without the need to compute alignments. For the problem of mapping RNA-seq reads to a reference transcriptome, this idea of replacing traditional alignment by faster mapping techniques is known to provide significant speed-ups [5–7]. We adapted to our case of mapping RNA-seq reads to a reference protein database, one such mapping technique, called quasi-mapping, the main idea of which is to determine the mapping by rapid look-ups of sub-strings of a query sequence. We incorporated our quasi-mapping technique into a pipeline for differential analysis. We show that our pipeline is > 1000× faster while remaining to be more accurate than assembly-based approach. Compared against other alignment/mapping strategies, we show that our method is fastest at the cost of sensitivity.

An implementation of our pipeline SAMAR-lite is available at https://github.com/bioinfodlsu/samar_lite

## Methods

### Notations

For a string *S*, we use *S*[*i* : *j*] to denote the substring of *S* starting at position *i* and ending at position *j*. We use |*S*| to denote the length of *S*.

### Reference index construction

**Fig. 1:**
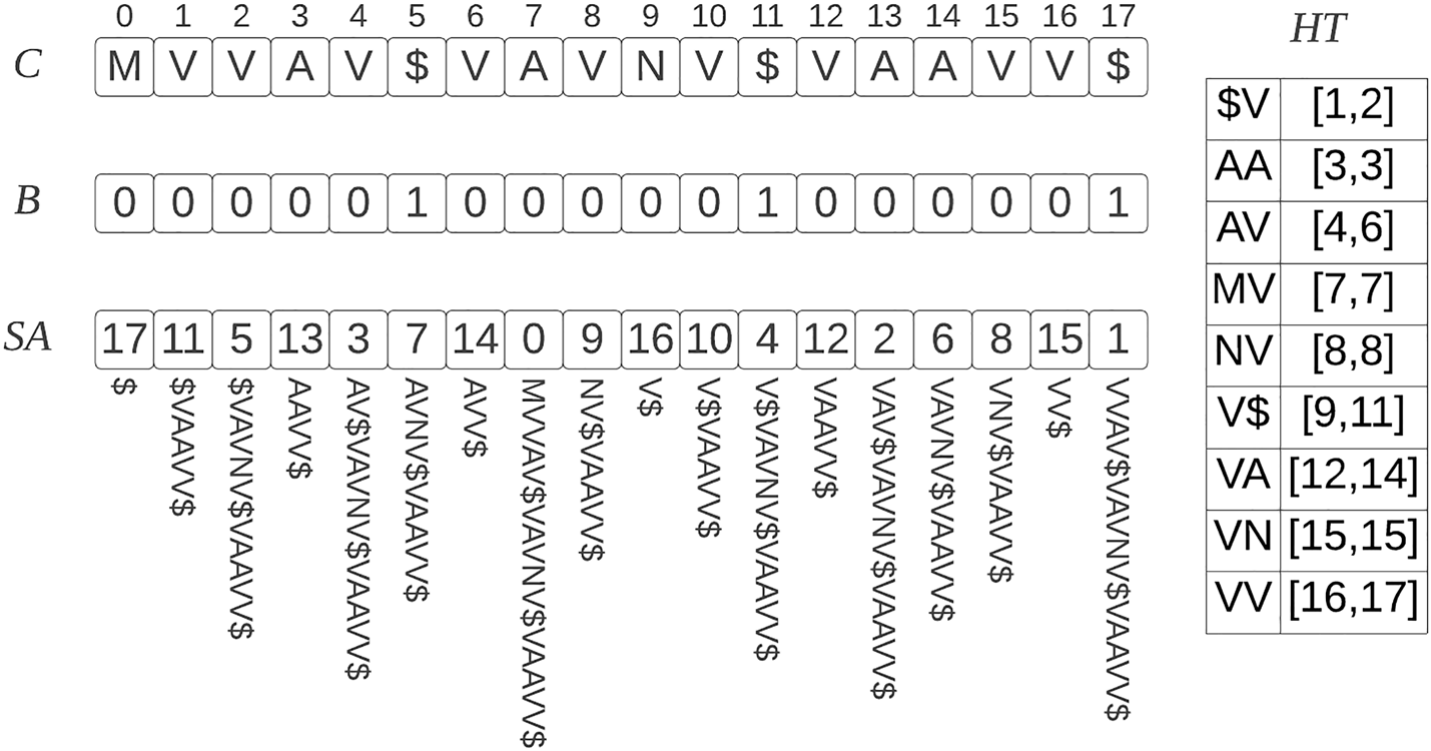
Example of the reference index data-structures for the set of sequences ${ \texttt {MVVAV, VAVNV, VAAVV}}$. *C* is the concatenated string, *B* is the bit-vector, *SA* suffix array, and *HT* is a hash table mapping a 2-mer to a suffix array interval containing suffixes whose prefix is the 2-mer

In the first stage of our method, we construct an index of the reference set of proteins to allow quick sub-string searches. We describe the data-structures below with the aid of an example shown in Fig. 1. We concatenate the reference sequences into a string *C*, separating the individual sequences by the special character $. We construct the suffix array *SA* of *C*, which is an array containing the indices of the sorted suffixes of *C*, and allows for fast sub-string searches using binary search. We augment *SA* with a hash table *HT* which maps a k-mer *S* present in *C* to *HT*(*S*) which is an interval in *SA* containing suffixes that have *S* as prefix. The role of *HT* is to allow faster searches in *SA* by reducing the search area of binary search. This idea has been explored in greater detail in [8]. Additionally, we construct a bit vector *B* of the same length as *C* containing 1 at each position corresponding to the special character $ and 0 elsewhere. A rank query $$rank_B(i)$$ takes a position *i* of *SA* as input and returns the number of 1s up to position *i* in *B*, essentially identifying the protein sequence in *C* corresponding to position *i* in the suffix array. A rank query can be accomplished in constant time by representing *B* using a succinct data-structure. A rank query can be generalized to $$rank_B([i,j])$$, where it takes an interval [*i*, *j*] of *SA* and returns the corresponding set of proteins.

### Quasi-mapping

In the second stage, we map each RNA-seq read to protein sequence(s) likely to be the translation product of the transcript from which the read originates. Our method closely follows the quasi-mapping technique of [6]. The core idea is to determine the mapping based on the set of k-mers and extended k-mer matches that are found rapidly using the data-structures described above.

In more detail, for each read, we first translate it into six amino acid sequences, one for each possible reading frame. Let *T* be one such translation. We iterate through the positions of *T*, performing at a position *pos*, the actions described below and also in the psuedo-code in Algorithm 1. If the k-mer starting at *pos* is a key in *HT*, we obtain its corresponding suffix array interval *sai*. We next attempt to extend the match by adding the amino acid at $pos+k$ to the current k-mer and performing a binary search within *sai*. We repeat this extension process until no matches can be found. At the end of the extension process, let $k'$ be the length of the extended k-mer and $sai'$ be the maximal suffix array interval containing suffixes whose prefix is $T[pos:pos + k'$]. Let $\mathbb {P}$ be the set of protein sequences whose sub-strings are in the interval $sai'$. For each $P \in \mathbb {P}$, we record that there was a $k'$-length substring match. The next iteration begins at $pos+k'$, or at $pos+1$ if the k-mer at *pos* was not in *HT* to begin with.

This mapping procedure is run six times, once for each possible reading frame. As the final mapping output, we return all protein sequences whose coverage, which is the total length of sub-string matches found, is larger than a pre-specified threshold, for any of the reading frames.

**Algorithm 1:**
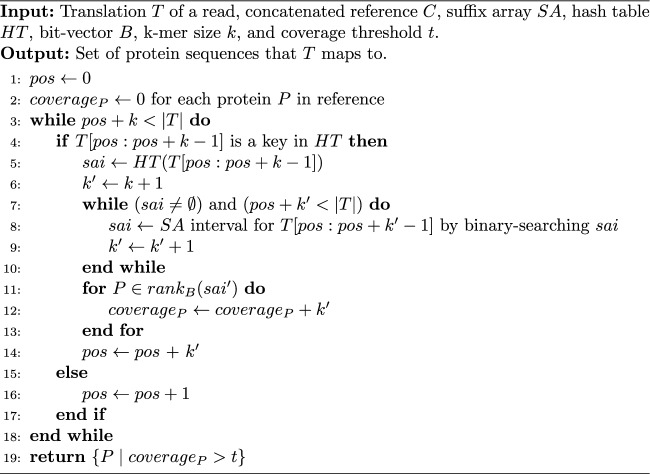
Quasi-mapping of one translation of a read. This process is repeated for all possible 6 frames.

### Counting

The mappings generated from the previous step are used to measure expression levels to be used for differential expression analysis. As in SAMAR, we follow the counting strategy of rescuing multi-mapping reads [9]. The counting is done in two passes. First, for each sequence *P* in the reference, we count the number of reads mapping uniquely to it, and normalize the count by |*P*|. Let this normalized count be $n_P$. Second, for each multi-mapping read, we update the count of each sequence *P* it multi-maps to, in proportion to $n_P$. That is, we add to $n_P$ the value $n_P / \sum \limits n_Q$, where the summation is over all *Q*s to which the read maps. If the denominator is zero, we distribute the count evenly among the proteins the read multi-maps to.

### Implementation details

We implemented the quasi-mapping method in the Rust programming language [10] using the RustBio library [11] for the suffix array and succinct representation of the bit vector for efficient rank queries. We incorporated the mapping step, the counting step, and DESeq2 [12] for differential analysis into a Snakemake [13] pipeline. The software is available at https://github.com/bioinfodlsu/samar_lite.

## Results

### Read simulation

For benchmarking, we used an RNA-seq read dataset that simulates a typical small-sample-size and low-coverage whole-genome gene expression experiment from our prior work [3]. The dataset was generated using Polyester [14] from the protein-coding transcriptome (BDGP6.28) of *D. melanogaster* obtained from Ensembl Genes 101 and containing 28,692 transcripts of 13,320 genes. We simulated two groups of read sets, with three replicates in each group. For the first group, we set the mean expression levels to be proportional to the FPKM values from an arbitrary poly-A+ enriched real RNA-seq data (ArrayExpress E-MTAB-6584). In the second group, around 30% of the transcripts were simulated to be differentially expressed with different levels of up-regulation and down-regulation. The transcripts were chosen by randomly selecting genes and setting only the highest expressing isoform to be differentially expressed. Each read set had roughly 20 million pairs of 100 bp reads with mean fragment length of 250 bp.

### Mapping performance and run time

#### Reference proteomes used

We first evaluated the performance of our method in the task of mapping the reads to four reference proteomes at varying levels of evolutionary divergence from the read source. The *D. melanogaster* proteome (Uniprot ID UP000000803) provides a baseline for performance comparison. The proteomes of *D. ananassae* (UP000007801) and *D. grimshawi* (UP000001070) were used to study the effect of mapping to the proteome of close relatives. Additionally, the proteome of *Anopheles gambiae* (UP000007062) was used to study the effect of mapping to a distant relative. Phylogenetic studies place *D. melanogaster* closer to *D. ananassae*, with a separation of 10-20 MY, than *D.grimshawi*, with separation of around 40 MY (see e.g. [15, 16]). According to one estimate, the lineages of *D. melanogaster* and *A. gambiae* separated roughly 250 MYA, and the average sequence identity at amino acid level in orthologous exons is only around 61.6% [17].

#### Tools compared

We compared our mapping performance against several existing tools for DNA-protein alignment/mapping of large data: LAST [18] (version 1060), DIAMOND [19] (version 2.0.12), and Kaiju [20] (version 1.9.0). We selected LAST as it was the alignment tool used in SAMAR [3]. We selected DIAMOND because it is a widely used DNA-protein alignment tool for big sequence data, although mainly focusing on metagenomic data. Kaiju, which employs maximum exact matches (MEM) to compute mappings, was originally proposed for metagenomic data, but it has recently been used by Seq2Fun [4] for RNA-seq dataset profiling. The commands used to run each tool is provided in the Appendix.

#### Evaluation metric

We evaluated mapping correctness as follows. Consider a read *r* from a transcript of *D. melanogaster* gene *g*. Let $M_g$ be the set of protein products of *g*. When using *D. melanogaster* proteome as reference, *r* was defined to be correctly mapped if at least one of the proteins it was mapped to, is in $M_g$. When using the other references, we determined correctness using pre-computed orthology maps between *D. melanogaster*–*D. ananassae* and *D. melanogaster*–*D. grimshawi* and *D. melanogaster* – *A. gambiae* pairs, which were obtained from InParanoid [21]. We defined *r* as being correctly mapped if at least one of the proteins it was mapped to is in an ortholog group containing a protein from $M_g$. A read with no mappings reported is not counted as correct or incorrect.

**Fig. 2:**
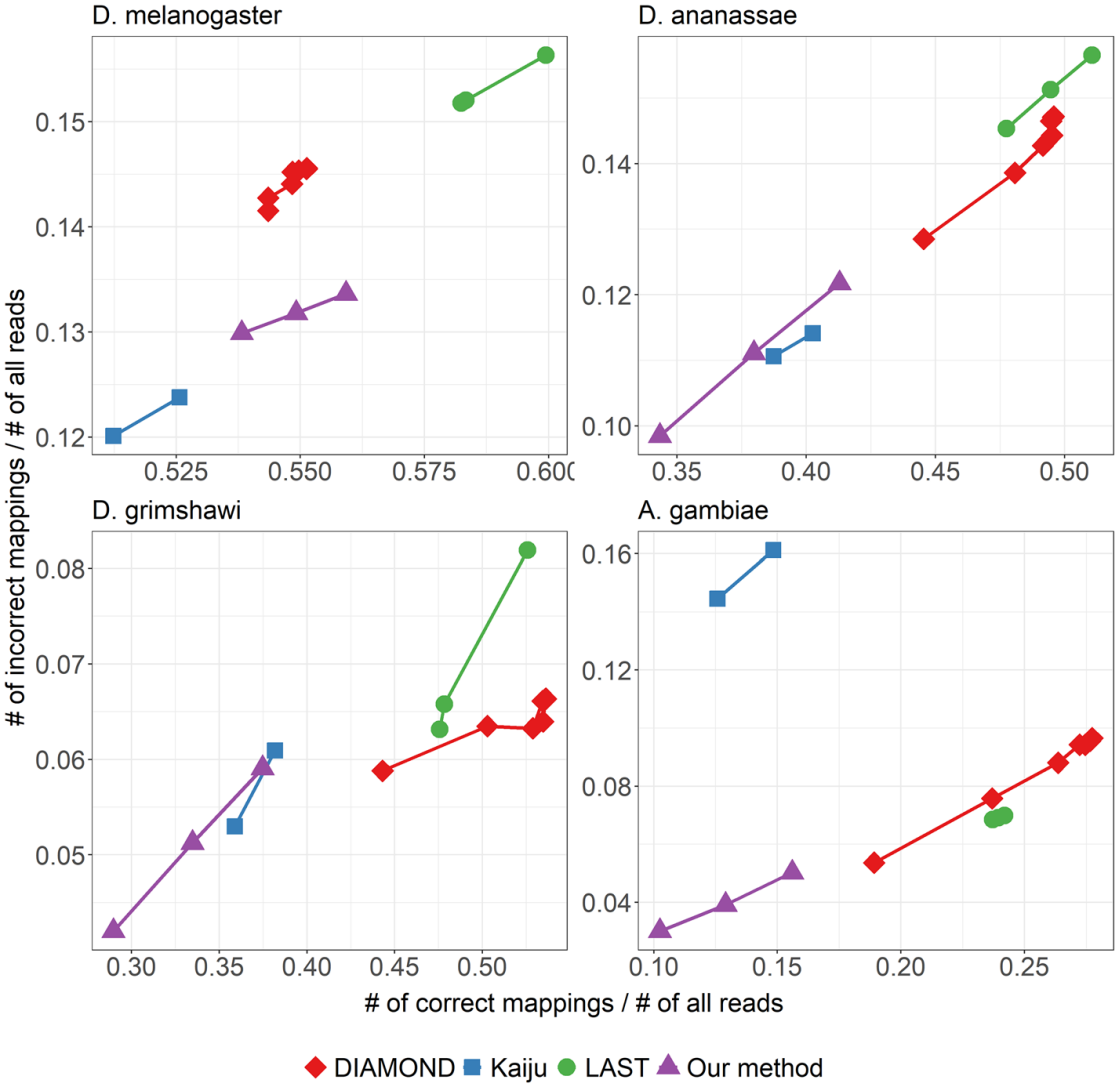
Mapping performance of the different aligners/mappers when using different reference proteomes. For DIAMOND, different points correspond to the different available presets: normal, fast, mid-sensitive, sensitive, more-sensitive, very-sensitive, and ultra-sensitive. For Kaiju, the points correspond to setting the mode to greedy and MEM. For LAST, the maximum mismap probability was set to 0.95,0.9, and 0.8. For our method, we used the k-mer size of 7 and coverage threshold was varied among 40, 50, and 60

**Fig. 3:**
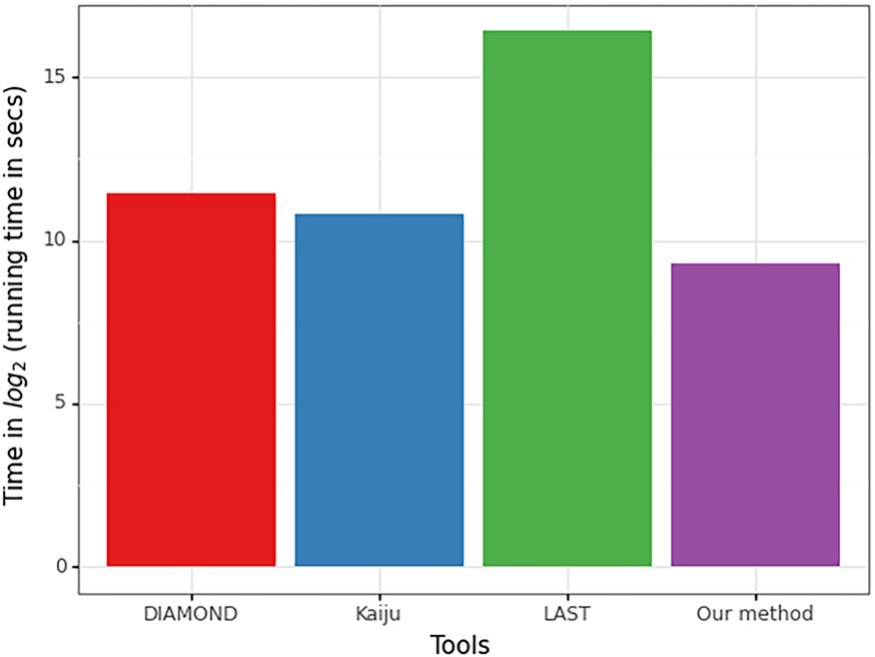
Comparison of mapping run times for a typical sample containing roughly 21 million pairs reads of length 100 bp each. Each tool was run on a single thread and with the following settings: normal for DIAMOND, greedy for Kaiju, maximum mismap probability of 0.95 for LAST, and k-mer 7 with coverage threshold 40 for our method

Mapping performance and running times are plotted in Figs. 2 and 3, respectively.

**Fig. 4:**
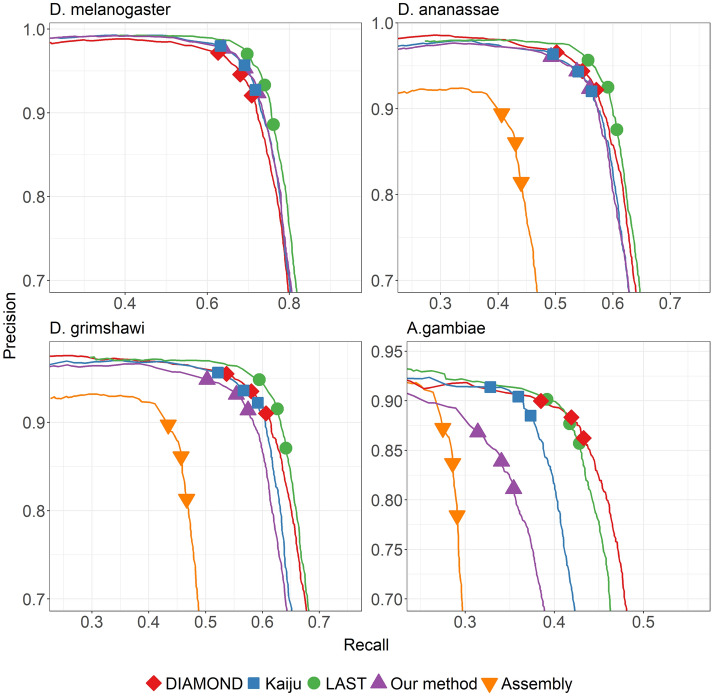
Precision-recall curves for differential gene expression analysis using different tools for the mapping step and when using different reference proteomes. The three shape markers in each curve correspond to setting the false discovery rates in DESeq2 to the values of 0.01, 0.05, and 0.1

### Differential expression analysis performance

Next, we fed the alignments/mappings produced by each tool to downstream differential expression analysis and compared their performance in identifying differentially expressed genes.

#### Pipelines compared

Of the possible parameters for each tool, we used the normal preset for DIAMOND, greedy for Kaiju, maximum mismap probability of 0.95 for LAST, and *k*-mer 7 with coverage threshold 40 for our method. The mappings were counted using the method described in the Methods section. For LAST, we used a slightly different variation offered in SAMAR, in which the counts are normalized by the length of the regions that have reads mapping to them rather than the entire length of the protein. The counts are then passed to DESeq2 [12] for differential expression analysis.

Additionally, to mimic the case of a non-model organism, we pretended that *D. melanogaster* reference transcriptome doesn’t exist; and we ran a typical assembly-based pipeline consisting of: Trinity [22] for de-novo transcriptome assembly, followed by Bowtie2 [23] for mapping the reads to the assembly, RSEM [24] for counting, tximport [25] for gene-level aggregation using the gene-to-transcript mapping provided by Trinity, and finally DESeq2 [12] for differential analysis. Dammit was used for annotating the assembled contigs against the *D. ananassae* and *D. grimshawi* proteomes. Since the annotation process produces many short local alignments, we keep only those alignments covering at least 50% of the length of the contig as evidence of homology. Commands used for assembly are described in the Appendix.

#### Evaluation metric

We evaluated each pipeline based on their precision and recall in predicting differentially expressed genes. Let $M_g$ be the set of protein products of a *D. melanogaster* gene *g*. When using the *D. melanogaster* reference, an actual up-regulated (down-regulated) gene *g* is defined as correctly predicted if at least one protein in $M_g$ (defined in previous section) was predicted to be up-regulated (down-regulated). When using the proteome of a relative as reference, *g* is defined as correctly predicted if at least one protein in an ortholog group containing $M_g$ was predicted to be up-regulated (down-regulated). We define precision as the proportion of predicted differentially expressed genes that are actually differentially expressed, and recall as the proportion of actual differentially expressed genes that were correctly predicted to be differentially expressed.

The results are shown in Fig. 4.

## Discussion

### Our method is > 1000× faster than assembly-based approach while being more accurate

We set out to build a quick-and-dirty differential gene expression analysis pipeline by avoiding transcriptome assembly and replacing a slower alignment step with quasi-mapping. As can be seen from the *D. grimshawi* and *D. ananassae* plots in Fig. 4, even when replacing sensitive full alignment step with the quasi-mapping step, our pipeline for identifying differentially expressed genes outperforms assembly-based approach. This gain comes with dramatic speed-up. Transcriptome assembly of a dataset containing a total of roughly 120 million pairs of reads, with 4 cores and 8 threads engaged took more than 7 days to complete. Our mapping takes less than 10 minutes for handling the same data, making it more than 100 times faster.

### When compared to other aligners/mappers, our method provides a trade-off between speed and sensitivity

As seen in the run-time plot of Fig. 3, our method runs the fastest among all the tools – being > 2.5× faster than the next fastest Kaiju, > 4× faster than DIAMOND, and > 100× faster than LAST. This might not be surprising as DIAMOND and LAST compute alignments using seed-and-extend approach, and LAST additionally computes appropriate alignment scoring scheme as well as alignment column probabilities. On the other hand, our method and Kaiju rely on finding exact matches. However, our speed-up comes at the cost of lesser mapping accuracy. As can be seen in Fig. 4, for the case of using the proteome of close relatives *D. ananassae* or *D. grimshawi* as reference, our method is overall less sensitive and precise than LAST and DIAMOND, while performing roughly similar as Kaiju. When using the proteome of the distant relative *A. gambiae*, the performance of our method worsens to the greatest degree compared to other aligners/mappers. The results of Fig. 4 essentially capture the differences in mapping performance shown in Fig. 2. There is a stark performance gap compared to LAST and DIAMOND, with our method correctly mapping 10–15% fewer reads than LAST or DIAMOND. Compared to Kaiju, the difference in mapping performance is not too apparent for close relatives, but Kaiju performs better when the reference becomes more distant.

We note that while we chose various reference proteomes to demonstrate the effect of varying levels of evolutionary divergence, a part of the differences we see in Figs. 2 and  4 might be attributed to the differences in the assembly and annotation pipeline employed to generate those reference sequences.

### Reduced alphabet

**Fig. 5:**
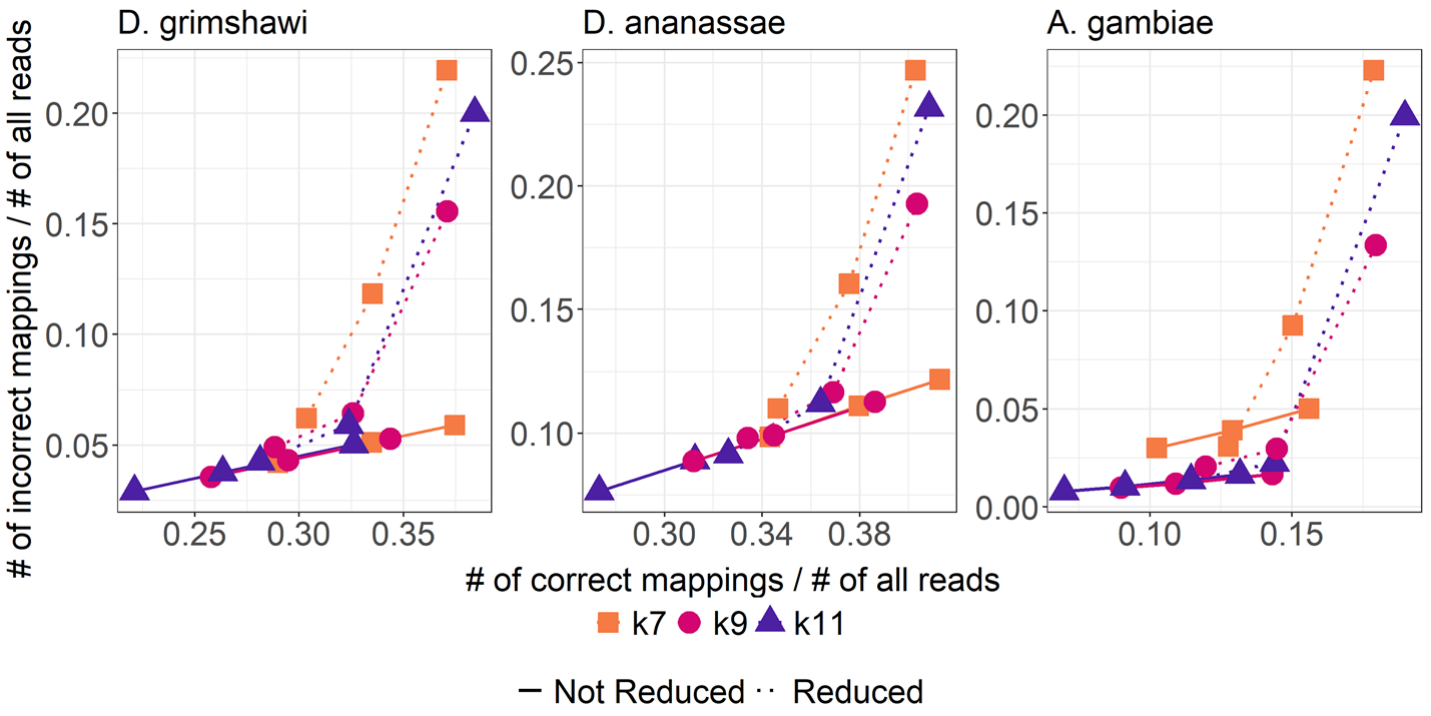
Comparing the performance of our method on the full amino acid alphabet versus a reduced one of size 11. For each curve, the three points from left to right correspond to coverage thresholds of 60, 50, and 40, respectively

We implemented quasi-mapping on the reduced amino-acid alphabet proposed by DIAMOND: {K,R,E,D,Q,N}, {C}, {G}, {H}, {I,L,V}, {M}, {F}, {Y}, {W}, {P}, {S,T,A}, where characters in the same set are treated to be equivalent. The results are shown in Fig. 5. We observed that for the reduced alphabet, coverage threshold value of 40 – corresponding to rightmost points in each curve – result in a substantial increase in incorrect mappings compared to the non-reduced alphabet. At the coverage threshold of 50, $k=11$ for reduced alphabet is close to the performance of $k=7$ for non-reduced alphabet, while being almost 2.5× faster.

### Effect of reference proteome

The availability of an accurate reference protein database is key to the performance of all methods evaluated in this paper, but more so for our method which relies on exact k-mer matches. This is seen, perhaps unsurprisingly, in our evaluations, where our method seems to be most sensitive to evolutionary divergence. Another factor that could affect performance is the presence of large number of highly similar sequences in the reference. This could be as a result of high level of recent gene/genome duplications, high amount of repetitive sequences in transcripts due to transposable elements, or because the reference spans multiple species and contains orthologs (e.g. UniProtKB). For all methods, it would be interesting to investigate the extent of performance degradation due to these factors at the level of mapping and in downstream functional analysis. As a practical remedy for the case of multi-species reference, it might be better to first reduce redundancy by running sequence clustering tools (e.g. CD-HIT [26] or MMSeq [27]) or find orthogroups (e.g. using OrthoFinder [28]).

### Future directions

It would be interesting to reduce the gap in sensitivity compared to traditional seed-and-extend methods without raising computational cost. Some promising direction include using spaced k-mers [29], syncmers [30], and minimally overlapping words [31]. Using spaced k-mers, for example, has been shown to be more sensitive than contiguous ones in other alignment-free sequence comparison applications [32], and are in fact also implemented in DIAMOND and LAST.

We also note that we chose the augmented suffix array data structure with memory usage not in mind, especially since our method uses negligible memory compared to de-novo transcriptome assembly. It might be interesting to explore other compact text indexes for cases where memory requirement is a concern.

## Conclusions

We have implemented a quick-and-dirty differential gene expression analysis pipeline for non-model organisms without a reference transcriptome. It uses quasi-mapping to rapidly map RNA-seq reads to a reference protein database, followed by a simple counting step the result of which can be fed to standard differential analysis tools like DESeq2. It is computationally super light-weight compared to the conventional approach of first building a transcriptome assembly. The quasi-mapping step itself is fast compared to other alignment/mapping techniques, but there is room for improvement in its sensitivity.

## Data Availability

Project name: Samar-lite, Project home page: https://github.com/bioinfodlsu/samar_lite, Operating system(s): Linux, macOS, Programming Language: Rust, Other requirements: Snakemake, Conda, License: MIT Licence, Any restrictions to use by non-academics: None, Datasets for benchmarking were obtained from public repositories: transcripts of protein-coding genes were obtained from the fruit fly assembly BDGP6.28 in Ensembl Genes 101; reference proteomes of *D. melanogaster* (UP000000803), *D. grimshawi* (UP000001070), *D. ananassae* (UP000007801), and *A. gambiae* (UP000007062) were obtained from UniProt.

## Appendix 1: Commands used for benchmarking aligners/mappers

All aligners/mappers were run on a system with an Intel i7-4710 CPU with 4 cores and 8 threads, and 32GBytes of RAM.

### Our method (SAMAR-lite)

The reference index was constructed using:

ref-align reference.fa 7

where 7 is the k-mer size. This was followed by mapping with a specified coverage threshold, e.g. 40 below :

alignr reference_7.json query.fastq 40 output

This is followed by counting:

python seq_count.py reference.fa input output

This ends with DESeq2, which performs differential expression analysis on the counts:

### LAST 1060

The reference index was constructed using:

lastdb -p index reference.fa

This was followed by aligment scoring scheme training: where sample.fa contains 6 amino-acid sequences (one each for a translation frame) per read of a sample of the input reads in fastq format.

This was followed by alignment:

This was followed by counting taken from the SAMAR [3] pipeline and DESeq2.

### DIAMOND 2.0.12

The reference index was constructed using:

diamond makedb –in reference.fa -d reference

This was followed by the alignment:

diamond blastx -q query.fa -d reference -o out.tsv

This was followed by counting, similar to the counting of our method, and DESeq2.

### Kaiju 1.9.0

The reference index was constructed using: Where n is the number of threads, a is the alphabet, and o is the output Burrows-Wheeler transform to be converted to an FM-index in the next command.

This was followed by mapping:

kaijux -f reference -i query.fastq -o output.kaiju

This was followed by counting, similar to the counting of our method, and DESeq2.

## Appendix 2: Commands used for benchmarking an assembly-based pipeline

### Assembly-based approach

De-novo transcriptome assembly was computed from all the reads in the dataset using Trinity (version 2.8.5): The reads were aligned to the assembled transcripts using Bowtie2 (version 2.4.1): Counting was done using RSEM: Transcript-level counts were aggregated at the gene level using tximport, based on the gene-transcript map constructed by Trinity, and finally differential expression analysis was performed using DEseq2.

Annotation was done against the reference proteome using Dammit.

dammit annotate Trinity.fasta –quick –user-databases dro_me_ref.fa -e 1e-10

Of the alignments that were reported in Trinity.fasta.x.dro_me_ref.fa.crbl.csv, we kept only those that cover at least 50% of the contig length.

## Abbreviations

SA: Suffix array
HT: Hash table
FPKM: Fragments per kilobase million
